# Supplementary figures and images for: Daily natural gas load prediction method based on APSO optimization and Attention-BiLSTM
Source: PeerJ Comput Sci. 2024 Feb 29;10:e1890. doi: 10.7717/peerj-cs.1890 (PMC10909168; doi:10.7717/peerj-cs.1890)

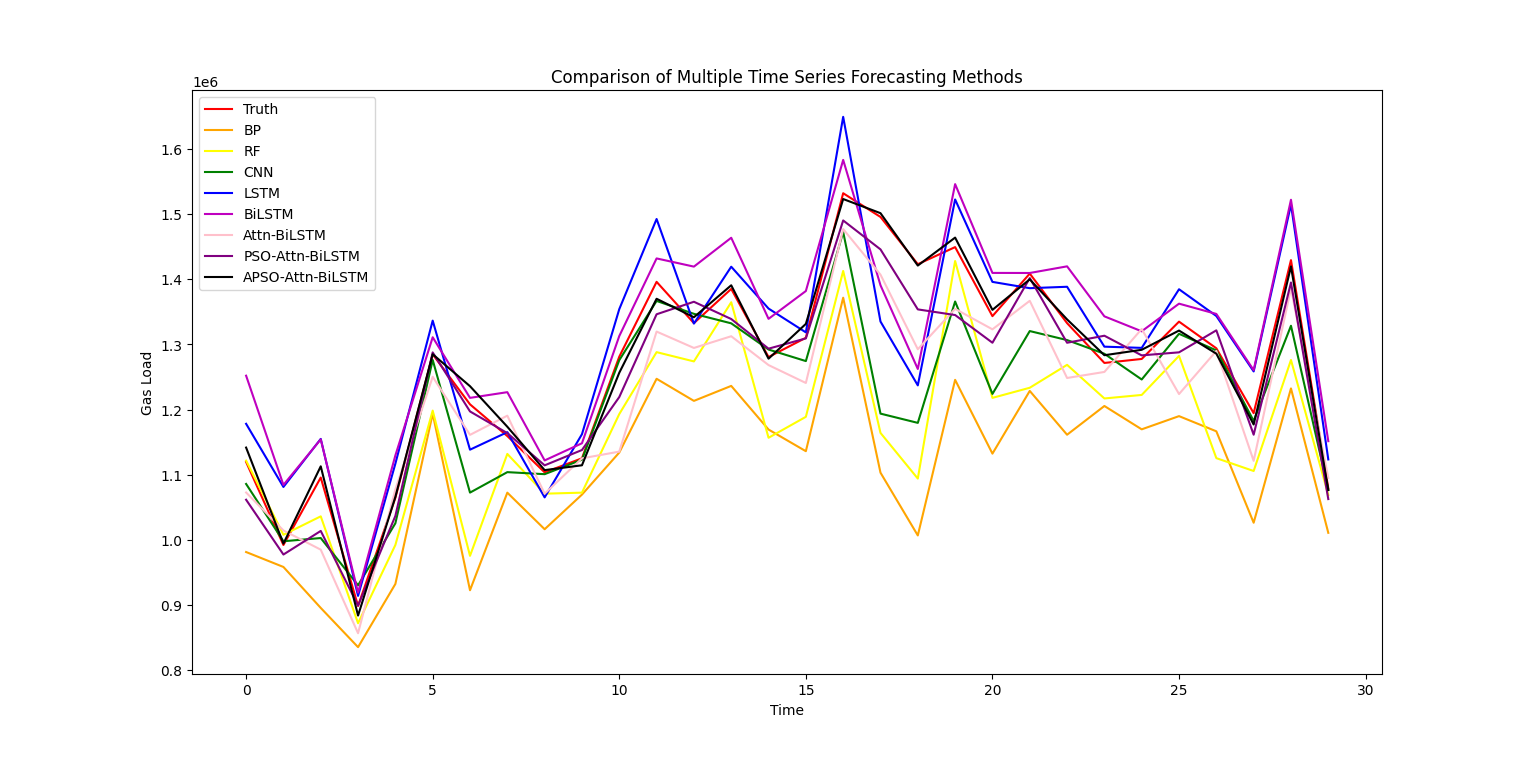

Supplement: Supplemental Information 1 — The prediction data show that the proposed model has high prediction accuracy. [file peerj-cs-10-1890-s001.zip › Code/Performance comparison chart.png]
